# Improving Medical Education: A Narrative Review

**DOI:** 10.7759/cureus.18773

**Published:** 2021-10-14

**Authors:** Tilak Joshi, Pravash Budhathoki, Anurag Adhikari, Ayusha Poudel, Sumit Raut, Dhan B Shrestha

**Affiliations:** 1 Department of Internal Medicine, Mount Sinai Hospital, Chicago, USA; 2 Department of Internal Medicine, BronxCare Health System, Bronx, USA; 3 Intensive Care Unit, Nepal Korea Friendship Municipality Hospital, Madhyapur Thimi, NPL; 4 Department of Emergency Medicine, Alka Hospital Pvt. Ltd., Kathmandu, NPL; 5 Department of Internal Medicine, Kathmandu Medical College, Kathmandu, NPL

**Keywords:** students, physicians, patient care, communication, medical education, professionalism, problem-based learning

## Abstract

With the advancement in basic and clinical sciences, medical education is also constantly evolving. The Accreditation Council for Graduate Medical Education (ACGME) has endorsed six core competencies to improve teaching and learning. This narrative review was conducted after searching the article databases (PubMed, PubMed Central, Embase, and Scopus) about the core competencies such as medical knowledge (problem-based learning), interpersonal communication, patient care, professionalism, practice-based learning and improvement, and system-based care endorsed by ACGME. We included randomized and quasi-experimental trials, cohorts, and case-control studies in this narrative review. In a problem-based learning modality, a real-life scenario is allocated to a group of students. Studies have shown that it is more effectively demonstrated by a better post-test score, improved concentration, and application of knowledge. Interpersonal communication skills promote collaboration with interdisciplinary teams, work quality, and patient adherence to treatment. Professionalism is a human attribute that creates a pleasant work environment and is an essential trait that improves patients' adherence to treatment. In system-based care, patients are benefitted through a well-structured plan of care. Finally, in practice-based learning, medical trainees learn to systematically evaluate the pattern of care and practice the best modality to improve the overall patient care and physician satisfaction. These core competencies need to be incorporated into all levels of medical training.

## Introduction and background

Medical education is ever-evolving because of constant advancements in basic sciences, clinical skills, and professionalism. As a result, it has changed drastically over the last two decades [1]. In 1999, the Accreditation Council for Graduate Medical Education (ACGME) endorsed a set of competencies required in every practicing physician. The six ACGME core competencies are professionalism, patient care, medical knowledge, interpersonal and communication skills, system-based care, and practice-based learning and improvement. Recently, the ACGME has developed the milestones as the roadmap of growth and development based on the core competencies [2]. We intend to provide an up-to-date narrative review in each core competency.

Medical knowledge acquisition and retention is a challenge that the traditional lecture-based teaching method has not addressed. The problem-based learning (PBL) approach was first designed and carried out at McMaster University in Hamilton, Ontario, Canada, in 1969. Its main goal was to promote active learning among students through self-directed and group-based learning. This method stood ahead of the traditional concept of a teacher as the primary educator. The PBL format is widely used in medical schools, where students are allocated into small groups and are provided with cases that resemble real-life scenarios. Students are involved in self-directed learning followed by group discussions, which a tutor accompanies [3].

Interpersonal communication is a vital part of medical education, where health professionals from various departments collaborate to enhance the learning curve and promote patients’ overall health status. Incorporating interpersonal communication in medical education is vital for preparing trainees to work in interdisciplinary teams [4]. Patient care is a broad term but essential to consider while acquiring medical education and clinical skills. For example, the interdisciplinary multimodal pain rehabilitation (IMPR) approach collaborates among health professionals involved in patients’ physical and psychosocial wellness and educating them about their diseases and treatment strategies. Subsequently, patient care is fostered with this systematic and meticulous approach [5].

Professionalism is a critical clinical discipline that every health personnel must follow from the beginning of their medical career. The belief that personality development is primarily an intrapersonal phenomenon supports the idea of professionalism as a collection of human traits and mutable attributes [6]. The Association of American Medical Colleges (AAMC) in 1991 has provided a set of examples that describe medical professionalism, which fellow health professionals have used to gauge their professionalism in medical education [7]. However, different institutions have different ways of assessing it.

Although several evidence-based care treatment plans are developed for diverse medical conditions, they cannot be executed without well-functioning multidisciplinary teams. A system-based care strategy involves planning, cooperation, and a structured management plan based on evidence that benefits patient care [8].

In practice-based learning and improvement, the trainees in medicine are expected to systematically investigate and evaluate their care to their patients and the practice patterns of their workplaces to identify the areas of improvement. They incorporate the best practices and implement the changes with the goal of improvement. They participate in educating patients, families, students, and other healthcare professionals [9].

All these sectors have a crucial effect on the upliftment of medical education.

## Review

Methods

We searched different databases such as PubMed, PubMed Central, Embase, and Scopus. Studies regarding problem-based learning, interpersonal communication, patient care, professionalism, practice-based learning and improvement, and system-based care were used for this review. Here, we have the narrative review to comply with the above terms.

Findings

Problem-Based Learning

Nine studies included in our problem-based learning review revealed its tremendous benefits. Most studies assessed this by conducting pre- and post-PBL sessions and pre- and post-traditional lecture-based sessions. Studies showed an increase in post-test scores after PBL sessions in comparison to team-based learning (TBL) [4,10]. This is further supported by the meta-analysis findings of Qin et al. and Galvao et al. [11,12].

In contrast, a study done in Harvard Medical School demonstrated no significant difference among the students whose mean prior exam scores were above the median in either group [7]. Apart from the scores, PBL improved attention, application of knowledge, better use of time, and available resources compared to conventional forms of learning [1,4,5,11,12]. Likewise, 97% of the students strongly agreed that PBL sessions should be part of the normal curriculum [11]. Due to the effect of the current pandemic, web-based learning has also become popular among medical students. Virtual learning was found to be as effective as face-to-face interaction learning [13].

Among nine studies on problem-based learning, four were from the USA, three from Europe, and one each from Africa and India. Five studies were from the medical profession, one each from nursing, dental, and physician assistant. Six studies enrolled undergraduate level, two including residents, and one enrolling postgraduate level. Three studies were randomized controlled trials (RCTs), two each cohort, a pre- and post-test single-group study, one each quasi-experimental trial, and a case-control study. A summary of some of the studies on problem-based learning is mentioned in Table 1 [10,13-20].

**Table 1:** Summary of various studies regarding problem-based learning. PBL = problem-based learning; TBL = team-based learning; E-PBL = electronic problem-based learning; CKD = chronic kidney disease; CD = Crohn's disease; MCQ = multiple choice question; UTI = urinary tract infection; SD = standard deviation.

S. No.	Study ID	Country	Experimental (PBL/TBL)	Control (Traditional learning)	P-value
1	Alaagib et al. (2018) [14]	Sudan	Physiology: 7.95 ± 1.65/10 (Mean ± SD/ Total marks) (N = 101)	Physiology: 9.68 ± 2.59/10 (Mean ± SD/Total marks) (N = 146)	p < 0.001
			Respiratory: 9.68 ± 2.59/20 (Mean ± SD/Total marks) (N =146)	Respiratory: 8.60 ± 4.02/10 (N = 146) (Mean ± SD/Total marks)	p = 0.006
2	Robson et al. (2009) [13]	United Kingdom	Mean score improvement in extended matching questions (EMQs) (E-PBL) (N = total students)	Not applicable	
			CKD = 0.009 (N = 23)		p = 0.998
			CD = 20.6 (N = 17)		p = 0.014
			UTI = 12 (N = 18)		p = 0.062
3	Smith et al. (2006) [15]	United States	Technical examination skills: (n = total students) Collaborative discovery (CD) = 10% (CI 4% to 17%) (n = 24)	Demonstration and practice (DP) = 12% (CI 6% to 19%) (n = 25)	p = 0.001
			Identifying key findings: CD = 5% (CI 2% to 9%)	Identifying key findings: DP = not significant	p = 0.004
4	Said et al. (2020) [16]	United States	Post session score = 9.71 ± 2.31 (Mean ± SD)	Pre-session score = 5.70 ± 1.88 (Mean ± SD)	p < 0.001
5.	Weidenbusch et al. (2019) [17]	Germany	Live case discussion (N = 30) (Mean ± SD)	Paper cases (N = 33) (Mean ± SD)	
			Pre-test = 5.34 ± 1.92		
			Post-test = 14.10 ± 3.32	Pre-test = 5.76 ± 2.24	
			Delayed knowledge application post-test = 3.36 ± 3.23		
			Subjective learning outcome = 4.20 ± 0.63	Post-test = 8.50 ± 2.44	
			Video-based discussion (N = 27) (Mean ± SD)		
			Pre-test = 4.76 ± 1.90	Delayed knowledge application post-test = 7.89 ± 2.41	
			Post-test = 11.69 ± 3.34		
			Delayed knowledge application post-test = 11.84 ± 2.92	Subjective learning outcome = 3.00 ± 0.99 (N = 31)	
			Subjective learning outcome = 3.18 ± 1.24		
6	Krupat et al. (2016) [18]	United States	Standardized final exam scores (PBL students): 47.13	Standardized final exam scores: 53.50	p = 0.36
			Mean final exam score: students with prior scores below the median of 64 = 26.88	Mean final exam score: students with prior scores below the median of 64 = 41.63	p = 0.05
7	Raupach et al. (2009) [19]	Germany	Final key feature examination mean score, (Mean ± SD): 31.9 ± 7.2 out of 55 points	Final key feature examination mean score (Mean ± SD): 31.7 ± 7.5 out of 55 points	p = 0.843
			Clinical case of chronic thromboembolic pulmonary hypertension (CTPH): T = 2.5 ± 1	Clinical case of CTPH: C = 2.0 ± 1.2	p = 0.003
			Summative MCQs: T = 50.3 ± 6.8	Summative MCQs: C = 50.3 ± 6.5	p = 0.973
8	Kelly et al. (2005) [20]	United States	PBL – engaged with each other = 74%	Lecture – engaged with each other = 9%	
			Engaged with teacher = 11%	Engaged with teacher = 58%	
			Self-engaged (reading/writing/not visibly interacting with others) = 15%	Self-engaged (reading/writing/not visibly interacting with others) = 33%	
9	Preeti et al. (2013) [10]	India	Mean marks obtained (N = 72) (Full marks: 20)		
			Pre-test score: 10.04		
			Post-test score: 14.89		

Interpersonal Communication

We included two studies under this heading. The first study was conducted in Switzerland to assess the triggers for conflict in a team and its impact on teamwork. Team members perceived that observed tensions were directly related to lower quality work based on multilevel regression analysis (except among anesthetists) with or without adjusting for hospital and surgery duration. Moreover, the quality of teamwork was rated high by all surgical team members [21]. The second study in Singapore showed increased self-confidence among the healthcare workers when they underwent a simulation-based interprofessional educational program for caring for a deteriorating patient [22]. Subsequently, a meta-analysis by Zolnierek et al. revealed marked improvement in patient adherence after the physician communication training. Furthermore, it was clearly stated that non-adherence was common among the patients attended by physicians who communicate poorly [23]. All these findings highlight the importance of interpersonal communication.

Summary of the studies done regarding interpersonal communication is presented in Table 2 [21,24].

**Table 2:** Studies regarding interpersonal communication.

S. No.	Study ID	Country	Interpersonal communication	Controls	p-value
1	Keller et al. (2019) [21]	Switzerland	Total surgeries observed (N = 137)	Tension-free surgeon heading a surgical team = 12	
			Hospital 1 (H1) N1 = 86		
			Hospital 2 (H2) N2 = 51		
			Mean duration between incision and closure: (mean ± standard deviation) = 3.67 ± 2.21 hours		
			H1 = 3.74 ± 2.43 hours		
			H2 = 3.55 ± 1.89 hours		
			Total tense communications = 340		
			Tensions per surgery (mean ± standard deviation) = 2.48 ± 5.19		
			Tensions per hour of surgery (mean ± standard deviation) = 0.57 ± 1.02		
			Tension free surgeries = 72 (52.6%)		
			Total surgeons = 30		
			Teamwork quality with tension = 4.87 on a 7-point scale	Teamwork quality without tension = 6.20 on a 7-point scale	
2	Liaw et al. (2014) [24]	Singapore	Medical students (N1 = 33), nursing students (N2 = 92), medical and nursing group		
			Self-confidence: Scale: 5-50, mean ± standard deviation = 34.26 ± 6.00	mean ± standard deviation = 27.68 ± 6.42	p = 0.001
			Perception: Scale: 8-40, mean ± standard deviation = 36.36 ± 3.46	Perception: Scale: 8-40, mean ± standard deviation = 31.90 ± 3.84	p = 0.001

Patient Care

Three studies in our review showed improved patient satisfaction and care after a collaborative and multi-modal approach in their management [5,25,26].

A study in the United States of America that focused on improving the quality of diabetes care in community centers was intervened by collaboration with community organizations, a self-management tool to track the patient’s progress, group cluster visits, and diabetes flow sheet. Of the respondents, 95% strongly believed that the collaboration was successful, and >80% wished to continue the interventions [25]. Similarly, a study in Sweden using a multi-modality approach to pain management for musculoskeletal pain found a significant improvement in patients' post-intervention status, which was sustained at the 12 months. However, the longer duration of the program was not found to be beneficial compared to shorter-duration programs [5]. A systematic review done by Hush et al. also exhibited that patient satisfaction is determined by interpersonal attributes and patient care approach. However, patient satisfaction was inconsistent with the treatment outcome [27]. Table 3 summarizes the findings of included studies.

**Table 3:** Summary of various studies included under “patient care.” HbA1c = glycosylated hemoglobin.

S. No.	Study ID	Country	Outcome		p-value
1.	Chin et al. (2004) [25]	United States	Matched patients (n = 969)		
			One HbA1c measurement: 1998: 80%	1999: 90%	<0.001
			Two HbA1c measurements at least 3 months apart: 1998: 37%	1999: 54%	<0.001
			Eye exam referral: 1998: 36%	1999: 47%	0.02
			Dietary counseling/referral to nutritionist: 1998: 51%	1999: 57%	0.10
			Foot exam/referral to podiatrist: 1998: 40%	1999: 64%	<0.001
			Dental referral: 1998: 7.2%	1999: 18%	0.001
			Lipid assessment: 1998: 55%	1999: 66%	0.02
			Urine microalbumin assessment: 1998: 13%	1999: 25%	0.001
			HbA1c value (%): 1998: 8.51%	1999: 8.32%	0.09
			All patients (n = 1628)		
			One HbA1c measurement: 1998: 80%	1999: 90%	<0.001
			Two HbA1c measurements at least 3 months apart: 1998: 38%	1999: 54%	<0.001
			Eye exam referral: 1998: 36%	1999: 47%	0.02
			Dietary counseling/referral to nutritionist: 1998: 49%	1999: 58%	0.04
			Foot exam/referral to podiatrist: 1998: 40%	1999: 64%	<0.001
			Dental referral: 1998: 6.7%	1999: 17%	<0.001
			Lipid assessment: 1998: 55%	1999: 67%	0.005
			Urine microalbumin assessment: 1998: 12%	1999: 25%	0.001
			HbA1c value (%): 1998: 8.52%	1999: 8.40%	0.30
2.	Tseli et al. (2020) [5]	Sweden	Short-form Health Survey (SF-36) Physical Component Summary (PCS) (0-100)	Post-Interdisciplinary Multimodel Pain Rehabilitation (Post IMPR)	
			Short: Baseline 27.9	Post IMPR 30.2	
			Medium: Baseline 28.6	Post IMPR 31.6	<0.001
			Long: Baseline 29.3	Post IMPR 29.7	0.335
			SF-36 Mental Component Summary (MCS) (0-100)		
			Short: Baseline 36.2	Post IMPR 39.6	
			Medium: Baseline 33.8	Post IMPR 38.6	0.084
			Long: Baseline 33.7	Post IMPR 39.5	0.853
			Pain intensity last 7 days		
			Short: Baseline 7.0	Post IMPR 6.2	
			Medium: Baseline 7.0	Post IMPR 6.1	0.166
			Long: Baseline 6.8	Post IMPR 6.2	0.891
			Euro-Qol 5-dimensions (EQ-5D index) (-0.594-1)		
			Short: Baseline 0.25	Post IMPR 0.35	
			Medium: Baseline 0.22	Post IMPR 0.37	0.313
			Long: Baseline 0.28	Post IMPR 0.34	0.632
			Hospital Anxiety and Depression Scale (HADS) A (0-21)		
			Short: Baseline 8.8	Post IMPR 7.9	
			Medium: Baseline 9.9	Post IMPR 8.0	0.528
			Long: Baseline 29.3	Post IMPR 29.7	0.941
			HADS A (0-21)		
			Short: Baseline 8.5	Post IMPR 6.5	
			Medium: Baseline 9.0	Post IMPR 6.6	0.519
			Long: Baseline 8.9	Post IMPR 6.7	0.293
3.	Schiff et al. (2017)[26]	United States	Rates of potential patient safety risks: Pre-intervention: 155 per 1000 patients with an abnormal lab value	Post-intervention: 54 per 1000 patients with abnormal lab value	(95% CI 0.24-0.50)
			Rates of serious patient safety risks: Pre-intervention: 28 per 1000 patients with an abnormal lab value	Post-intervention: 13 per 1000 patients with abnormal lab value	(95% CI 0.22–0.98)

Professionalism

A total of five studies qualified for the study under the heading of “professionalism” and are summarized in Table 4 [28-31]. Most health professionals are well aware of professionalism and its impact on medical education and clinical practice [31]. In a study in Japan, emergency medicine (EM) residents scored higher than EM physicians when questioned about confidentiality and sexual harassment [32]. Another study showed that clinical groups of students scored higher than preclinical students in a quiz about professionalism. However, the finding was not statistically significant [29]. In a study done in Australia, 95% of respondents stated that personal and professional development (PPD) helped them learn about professional development. Interviewing patients in the community and writing from the patient’s perspective helped students understand the biopsychosocial aspect of medicine and guided appropriate behavior in clinical practice [6]. Health professionals who are well informed about work habits and work ethics will foster patient adherence and create a healthy working environment.

**Table 4:** Summary of various studies regarding professionalism. IQR = interquartile range.

S. No.	Year	Country	Outcome			p-value
1.	Wiecha et al. (2008) [28]	United States	Identifying factors contributing to patient non-compliance control: number (%)		Intervention group: number (%)	
			Decreased 9 (8.8%)		Decreased 5 (4.5%)	
			No change 43 (42.2%)		No change 29 (25.9%)	
			Gained 50 (49.0%)		Gained 78 (69.6%)	
			Integrating patient’s cultural beliefs about health into your care of that patient control: number (percentage)		Intervention group: number (percentage)	
			Decreased 11 (10.7%)		Decreased 6 (5.4%)	
			No change 41 (39.8%)		No change 25 (22.3%)	
			Gained 51 (49.5%)		Gained 81 (72.3%)	
			Eliciting how a patient has been emotionally impacted by an illness control: number (percentage)		Intervention group: number (percentage)	
			Decreased 14 (13.5%)		Decreased 8 (7.1%)	
			No change 42 (40.4%)		No change 28 (25.0%)	
			Gained 48 (46.2%)		Gained 76 (67.9%)	
2.	Shiga et al. (2020) [32]	Japan	Frequency of the best or second-best responses for scenarios presented by residents and faculty: number (percentage)		Number (percentage)	
			Gifts: Total (T) = 153 (88.9%); Resident = 73 (87.9%)		Faculty = 80 (89.8%)	0.81
			Conflict of interest: T = 154 (89.7%); Resident = 72 (86.7%)		Faculty = 82 (92.2%)	0.32
			Confidentiality: T = 121 (69.9%); Resident = 64 (77.1%)		Faculty = 56 (62.9%)	0.048
			Impairment: T = 145 (84.3%); Resident = 67 (80.7%)		Faculty = 78 (87.6%)	0.29
			Harassment: T = 77 (44.5%); Resident = 36 (43.3%)		Faculty = 41 (46.1%)	0.76
			Honesty: T = 151 (87.3%); Resident = 76 (81.7%)		Faculty = 74 (83.1%)	0.11
3.	Haque et al. (2016) [29]	Malaysia	Professionalism according to sex: mean (SD)			
			Honesty: Male: 22.39 (3.69)	Female: 22.33 (3.26)		0.956
			Accountability: Male: 17.82 (2.70)	Female:18.69(3.12)		0.72
			Confidentiality: Male 15.31 (2.28)	Female: 15.61(3.42)		0.531
			Respectful: Male 24.00 (3.09)	Female: 24.76(3.14)		0.190
			Responsibility: Male 22.35 (2.99)	Female 23.58(2.82)		0.020
			Compassion: Male 15.76 (2.64)	Female 16.99(5.98)		0.130
			Communication: Male 18.06 (3.28)	Female 19.10(3.00)		0.055
			Maturity: Male 23.39 (3.66)	Female 24.04 (2.85)		0.248
			Self-directed learning: Male 7.88 (1.29)	Female 8.24 (1.51)		0.086
			Grand total score: Male 166.98 (20.15)	Female 173.34 (18.09)		0.61
			Comparison of mean score of professionalism by medical students according to educational phase: mean (SD) total possible score: 220			
			Honesty: Preclinical: 22.67 (3.52)	Clinical: 22.11 (3.34)		0.361
			Accountability: Preclinical: 18.02 (2.48)	Clinical: 18.62(3.31)		0.195
			Confidentiality: Preclinical 15.44 (3.47)	Clinical: 15.54(2.67)		0.762
			Respectful: Preclinical 24.19 (3.25)	Clinical: 24.68(3.04)		0.234
			Responsibility: Preclinical 23.33 (2.82)	Clinical:23.14(3.04)		0.676
			Compassion: Preclinical 16.36 (2.50)	Clinical: 16.64(6.29)		0.733
			Communication: Preclinical 18.36 (3.39)	Clinical: 18.97(2.93)		0.179
			Maturity: Preclinical 23.01 (3.42)	Clinical 23.70 (3.01)		0.918
			Self-directed learning: Preclinical 8.15 (1.41)	Clinical: 8.12 (1.45)		0.923
			Grand total score: Preclinical 170.17 (18.67)	Clinical: 171.49 (19.49)		0.694
			Male: 166.98 ± 20.15	Female: 173.34+/-18.09		NS
			Preclinical: 170.17 ± 18.67	Clinical: 171.49+/-19.49		NS
5.	Curran et al. (2020) [30]	Canada	Friedman analysis of peer assessment score improvement over time; Work Habits Score (TP = time point)			<0.001
			Mean: TP1 26.25; TP2 26.5; TP3 26.93; TP4 27.45; Friedman X2 (3) = 52.07			
			Mean Rank: TP1 1.95; TP2 2.22; TP3 2.50; TP4 3.33			
			Interpersonal Habits Score			<0.001
			Mean: TP1 28.19; TP2 28.56; TP3 28.83 TP4 29.05; Friedman X2(3) = 56.23			
			Mean Rank TP1 1.73; TP2 2.38; TP3 2.75; TP4 3.15			
6.	Byakika-Kibwika et al. (2015) [31]	Uganda	Duration in teaching service: median: 39; IQR 32.7-43.2			
			Doctorate degree: 7 (22%)			
			Master’s degree: 23 (72%); Fellowship: 1 (3%)			
			Bachelor’s degree 1 (3%)			

System-Based Care

Multiple studies have shown that system-based care aligns with the quality of patient care and decreases the risks associated with the clinical practice [8,33]. In addition, inter-specialty collaboration to manage hard and soft tissue injuries with a systematic approach has also boosted the confidence and reinforced their surgical skills [34]. For example, a study undertaken in the United States implementing the Six Building Blocks Program for managing patients taking opioids for chronic pain analyzed its effect on the work-life of primary care providers and staff. As a result, the involved staff reported improved work-life balance, confidence and comfort in clinical areas, ease in managing cases with chronic pain, increased comfort in work processes and their role, and increased collaboration after implementing the program [33]. Thus, system-based care has a two-way advantage where the patient gets better care along with physician satisfaction. Table 5 summarizes the study findings.

**Table 5:** Summary of studies regarding system-based care. HbA1c = glycosylated hemoglobin; LDL = low-density lipoprotein; BP = blood pressure.

S. No.	Year	Country	Outcome	
1.	Milne et al. (2020) [34]	United Kingdom	Before course:	Reasons for inability to treat facial and oral wounds in the emergency department:
				Service pressures
			25/48 participants: confident to repair lip laceration in an adult either with supervision or independently	Lack of confidence in the ability
			15/48 participants: confident to repair full-thickness laceration of the pinna	Inability to provide adequate analgesia (including sedating children)
2.	Stevens et al. (2010) [8]	United States	Clinical outcomes for 15 teams in California Academic Chronic Care Collaborative June 2007	Clinical outcomes for 15 teams in California Academic Chronic Care Collaborative May 2008
			HbA1c < 7%, total registry size (n = 1302); weighted average 42.4	HbA1c < 7%, total registry size (n = 1559); weighted average 44.7
			LDL < 100 mg/dL, total registry size (n = 1034); weighted average 50.9	LDL < 100 mg/dL, total registry size (n = 1351); weighted average 59.5
			BP < 130/80, total registry size (n = 1302); weighted average 36.4	BP < 130/80 total registry size (n = 1559); weighted average 47.4
			Retinal exam total registry size (n = 1178); weighted average 25.5	Retinal exam total registry size (n = 1437); weighted average 41.1
			Foot exam total registry size (n = 1178); weighted average 30.4	Foot exam total registry size (n = 1437); weighted average 56
			Documented self-management goal total registry size (n = 1300); weighted average 10.7	Documented self-management goal total registry size (n = 1559); weighted average 41.4

Practice-Based Learning and Improvement

Practice-based learning and improvement (PBLI) connect continuous learning to good patient care. It is an experiential continuum that reveals trainees their own learning needs and the needs of their practices. The trainees then develop and implement plans for their self-improvement and the improvement of their practices. Small and sustained changes in individual clinicians and practice patterns can result in the improvement of healthcare systems [35]. For example, a study by Ogrinc et al. in 2004 revealed that four weeks of PBLI elective by internal medicine residents improved quality Improvement Knowledge Application Tool scores compared to the control group [36]. Other studies by Varkey et al. in 2009 describe that the application of PBLI and systems-based practice in the curriculum in Mayo Clinic resulted in a 13% increase in perceived ability to measure competency in systolic blood pressure (SBP), no change in their perceived ability to measure competence in PBLI, a 15% increase in their ability to provide written documentation of competence in PBLI and a 35% increase in their ability to provide written documentation of competence in SBP between 2005 and 2007. In addition, 70% of the residents participated in quality improvement (QI) projects during the time [37]. Therefore, it is crucial to develop and implement a curriculum in PBLI by every teaching medical institution.

## Conclusions

Improvement of medical education involves integrating problem-based learning, robust interpersonal communication, patient care, professionalism, and practice-based learning and improvement and improving system-based care. Incorporating and implementing these core competencies in medical curricula is essential in all levels of medical training, including undergraduate level, residency, and fellowship training.

## References

[1] Patel M (2016). Changes to postgraduate medical education in the 21st century. Clin Med (Lond).

[2] Edgar L, McLean S, Hogan SO, Hamstra S, Holmboe ES (2020). The Milestones Guidebook. https://www.acgme.org/globalassets/milestonesguidebook.pdf.

[3] Karimi R (2011). Interface between problem-based learning and a learner-centered paradigm. Adv Med Educ Pract.

[4] Boshoff K, Murray C, Worley A, Berndt A (2020). Interprofessional education placements in allied health: a scoping review. Scand J Occup Ther.

[5] Tseli E, LoMartire R, Vixner L, Grooten WJ, Gerdle B, Äng BO (2020). What Is the effectiveness of different duration interdisciplinary treatment programs in patients with chronic pain? A large-scale longitudinal register study. J Clin Med.

[6] Langendyk V, Mason G, Wang S (2016). How do medical educators design a curriculum that facilitates student learning about professionalism?. Int J Med Educ.

[7] Reimer D, Russell R, Khallouq BB, Kauffman C, Hernandez C, Cendán J, Castiglioni A (2019). Pre-clerkship medical students' perceptions of medical professionalism. BMC Med Educ.

[8] Stevens DP, Bowen JL, Johnson JK (2010). A multi-institutional quality improvement initiative to transform education for chronic illness care in resident continuity practices. J Gen Intern Med.

[9] Dressler DD, Pistoria MJ, Budnitz TL, McKean SC, Amin AN (2006). Core competencies in hospital medicine: development and methodology. J Hosp Med.

[10] Preeti B, Ashish A, Shriram G (2013). Problem based learning (PBL) - an effective approach to improve learning outcomes in medical teaching. J Clin Diagn Res.

[11] Qin Y, Wang Y, Floden RE (2016). The effect of problem-based learning on improvement of the medical educational environment: a systematic review and meta-analysis. Med Princ Pract.

[12] Galvao TF, Silva MT, Neiva CS, Ribeiro LM, Pereira MG (2014). Problem-based learning in pharmaceutical education: a systematic review and meta-analysis. ScientificWorldJournal.

[13] Robson J (2009). Web-based learning strategies in combination with published guidelines to change practice of primary care professionals. Br J Gen Pract.

[14] Alaagib NA, Musa OA, Saeed AM (2019). Comparison of the effectiveness of lectures based on problems and traditional lectures in physiology teaching in Sudan. BMC Med Educ.

[15] Smith CA, Hart AS, Sadowski LS (2006). Teaching cardiac examination skills. A controlled trial of two methods. J Gen Intern Med.

[16] Said JT, Thompson LL, Foord L, Chen ST (2020). Impact of a case-based collaborative learning curriculum on knowledge and learning preferences of dermatology residents. Int J Womens Dermatol.

[17] Weidenbusch M, Lenzer B, Sailer M (2019). Can clinical case discussions foster clinical reasoning skills in undergraduate medical education? A randomised controlled trial. BMJ Open.

[18] Krupat E, Richards JB, Sullivan AM, Fleenor TJ Jr, Schwartzstein RM (2016). Assessing the effectiveness of case-based collaborative learning via randomized controlled trial. Acad Med.

[19] Raupach T, Muenscher C, Anders S, Steinbach R, Pukrop T, Hege I, Tullius M (2009). Web-based collaborative training of clinical reasoning: a randomized trial. Med Teach.

[20] Kelly PA, Haidet P, Schneider V, Searle N, Seidel CL, Richards BF (2005). A comparison of in-class learner engagement across lecture, problem-based learning, and team learning using the STROBE classroom observation tool. Teach Learn Med.

[21] Keller S, Tschan F, Semmer NK (2019). "Disruptive behavior" in the operating room: a prospective observational study of triggers and effects of tense communication episodes in surgical teams. PLoS One.

[22] Liaw SY, Zhou WT, Lau TC, Siau C, Chan SW (2014). An interprofessional communication training using simulation to enhance safe care for a deteriorating patient. Nurse Educ Today.

[23] Zolnierek KB, Dimatteo MR (2009). Physician communication and patient adherence to treatment: a meta-analysis. Med Care.

[24] Liaw SY, Ooi SW, Rusli KD, Lau TC, Tam WW, Chua WL (2020). Nurse-physician communication team training in virtual reality versus live simulations: randomized controlled trial on team communication and teamwork attitudes. J Med Internet Res.

[25] Chin MH, Cook S, Drum ML (2004). Improving diabetes care in Midwest community health centers with the health disparities collaborative. Diabetes Care.

[26] Schiff GD, Reyes Nieva H, Griswold P (2017). Randomized trial of reducing ambulatory malpractice and safety risk: results of the Massachusetts PROMISES project. Med Care.

[27] Hush JM, Cameron K, Mackey M (2011). Patient satisfaction with musculoskeletal physical therapy care: a systematic review. Phys Ther.

[28] Wiecha JM, Markuns JF (2008). Promoting medical humanism: design and evaluation of an online curriculum. Fam Med.

[29] Haque M, Zulkifli Z, Haque SZ (2016). Professionalism perspectives among medical students of a novel medical graduate school in Malaysia. Adv Med Educ Pract.

[30] Curran VR, Sharpe D, Forristall J, Flynn K (2008). Student satisfaction and perceptions of small group process in case-based interprofessional learning. Med Teach.

[31] Byakika-Kibwika P, Kutesa A, Baingana R (2015). A situation analysis of inter-professional education and practice for ethics and professionalism training at Makerere University College of Health Sciences. BMC Res Notes.

[32] Shiga T, Nakashima Y, Norisue Y (2020). Comparison of professionalism between emergency medicine resident physicians and faculty physicians: a multicenter cross-sectional study. PLoS One.

[33] Ike B, Baldwin LM, Sutton S, Van Borkulo N, Packer C, Parchman ML (2019). Staff and clinician work-life perceptions after implementing systems-based improvements to opioid management. J Am Board Fam Med.

[34] Milne S, Walshaw EG, Webster A, Mannion CJ (2020). Active learning in head and neck trauma: outcomes after an innovative educational course. [PREPRINT]. Br J Oral Maxillofac Surg.

[35] Lynch DC, Swing SR, Horowitz SD, Holt K, Messer JV (2004). Assessing practice-based learning and improvement. Teach Learn Med.

[36] Ogrinc G, Headrick LA, Morrison LJ, Foster T (2004). Teaching and assessing resident competence in practice-based learning and improvement. J Gen Intern Med.

[37] Varkey P, Karlapudi S, Rose S, Nelson R, Warner M (2009). A systems approach for implementing practice-based learning and improvement and systems-based practice in graduate medical education. Acad Med.

